# Research on machine vision and deep learning based recognition of cotton seedling aphid infestation level

**DOI:** 10.3389/fpls.2023.1200901

**Published:** 2023-08-14

**Authors:** Xin Xu, Jing Shi, Yongqin Chen, Qiang He, Liangliang Liu, Tong Sun, Ruifeng Ding, Yanhui Lu, Chaoqun Xue, Hongbo Qiao

**Affiliations:** ^1^ College of Information and Management Science, Henan Agricultural University, Zhengzhou, China; ^2^ Institute of Plant Protection, Xinjiang Academy of Agricultural Sciences, Urumqi, China; ^3^ Institute of Plant Protection, Chinese Academy of Agricultural Sciences, Beijing, China; ^4^ Zhengzhou Tobacco Research Institute of China National Tobacco Corporation (CNTC), Zhengzhou, China

**Keywords:** *Aphis gossypii* Glover, Faster R-CNN, YOLOv5, SSD, deep learning

## Abstract

*Aphis gossypii* Glover is a major insect pest in cotton production, which can cause yield reduction in severe cases. In this paper, we proposed the *A. gossypii* infestation monitoring method, which identifies the infestation level of *A. gossypii* at the cotton seedling stage, and can improve the efficiency of early warning and forecasting of *A. gossypii*, and achieve precise prevention and cure according to the predicted infestation level. We used smartphones to collect *A*. *gossypii* infestation images and compiled an infestation image data set. And then constructed, trained, and tested three different *A. gossypii* infestation recognition models based on Faster Region-based Convolutional Neural Network (R-CNN), You Only Look Once (YOLO)v5 and single-shot detector (SSD) models. The results showed that the YOLOv5 model had the highest mean average precision (mAP) value (95.7%) and frames per second (FPS) value (61.73) for the same conditions. In studying the influence of different image resolutions on the performance of the YOLOv5 model, we found that YOLOv5s performed better than YOLOv5x in terms of overall performance, with the best performance at an image resolution of 640×640 (mAP of 96.8%, FPS of 71.43). And the comparison with the latest YOLOv8s showed that the YOLOv5s performed better than the YOLOv8s. Finally, the trained model was deployed to the Android mobile, and the results showed that mobile-side detection was the best when the image resolution was 256×256, with an accuracy of 81.0% and FPS of 6.98. The real-time recognition system established in this study can provide technical support for infestation forecasting and precise prevention of *A. gossypii*.

## 1 Introduction 

Cotton is an important cash crop in China, with Xinjiang serving as the main production location. In 2022, the cotton planting area in Xinjiang was 2.4969 million hectares, accounting for 83.22% of China; the yield per unit area was 2158.9 kilograms per hectare; the total production was 5.391 million tons, accounting for 90.20% of China’s total production (National Bureau of Statistics, 2022). *Aphis gossypii* Glover is one of the most serious insect pests in cotton cultivation (Luo et al., 2017), and can cause leaf curling and wilting after sucking nutrients from cotton leaves, which in turn affects the growth and development of cotton plants, leading to a decrease in yield and quality. The occurrence of *A. gossypii* leads to a cotton yield reduction of approximately 15% to 30% and can cause total crop failure in severe cases (Herron et al., 2000; Fan et al., 2013). Therefore, it is important to study the *A. gossypii* occurrence patterns and infestation features and to explore a fast and efficient method for monitoring and detecting this pest. In this way, the efficiency of early warning and forecasting can be improved cotton yield reduction can be mitigated.

The current method of *A. gossypii* infestation detection is still mainly based on manual investigation, mainly through manual field inspection, measurement, statistics and expert identification (Liu et al., 2019), which is not only time-consuming and laborious but also subjective, with a high rate of misjudgment and poor real-time response. Thus, there is an urgent need for a time- and labor-saving technology for *A. gossypii* detection to improve the efficiency of infestation level recognition.

Image processing technology, in which image signals are converted into the corresponding digital signals and are processed by using computers, provides new technological solutions for the detection of crop pests and diseases(Arnal, 2013). Crop pest and disease image recognition technology has the characteristics of rapidity, accuracy and real-time employability. Research on this technology has mainly focused on three aspects: crop pest and disease image segmentation, feature extraction and classification recognition. Zhang et al. (2018) proposed an automatic image segmentation model for diseased leaves with active gradients and local information in which image details such as cotton leaves with a background of uneven illumination, shadows and weeds could be segmented to better achieve the ideal extraction of leaf edges. Lu and Ye (2020) proposed a semiautomatic locust species and age information detection model based on locust image segmentation, feature variable extraction and support vector machine classification, with 96.16% detection accuracy. Nabilah et al. (2020) used six traditional feature methods and six deep learning feature methods to extract significant pest features from chili leaf images, and the extracted features were fed into a support vector machine (SVM), random forest, and an artificial neural network for the recognition task. The results showed that the deep learning feature-based methods outperformed the traditional feature-based methods, and the best accuracy of 92.10% was obtained using the SVM classifier. Khan et al. (2020) designed a cucumber leaf disease detection and classification system and achieved 98.08% classification accuracy for five cucumber leaf diseases using a multi-class support vector machine (M-SVM) approach. Wang et al. (2019) proposed a pest detection and identification diagnosis system based on transfer learning, which was able to train and test 10 types of pests with an accuracy of 93.84%. Wang et al. (2020) proposed a convolutional neural network recognition model based on the Inception module and dilated convolution. By setting different parameters, six improved models were obtained, which were trained to recognize 26 diseases in 14 different crops. The final experiment could achieve an average recognition accuracy of 99.37%. Hu et al. (2020) proposed a convolutional neural network based on data augmentation combined with migration learning to recognize corn leaf diseases, and achieved an average recognition accuracy of 97.6% for Corn Gray leaf spot, Corn Common rust, Corn Northern Leaf Blight, and healthy leaves, with an accuracy of more than 95% for each category. To accurately identify small agricultural pests, Dong et al. (2021) proposed a CRA-Net which included a channel recalibration feature pyramid network and adaptive anchor module. The results showed that the method achieved an average precision of 67.9%, which was superior to other state-of-the-art methods. Gu et al. (2021) proposed a method for diagnosing plant diseases and identifying pests with deep features based on transfer learning, and the proposed model achieved 96.02% and 99.61% accuracy, respectively. To solve the problem of small pest identification and detection, Wang R. J. et al. (2021) proposed a sampling-balanced region proposal generation network, and designed a novel adaptive regionally of interest selection method to learn features at different levels of the feature pyramid. Several experiments on the proposed AgriPest21 data set showed that the method could achieve an average recall rate of 89.0% and mAP of 78.7%, superior to other state-of-the-art methods. Wei et al. (2022) proposed a multiscale feature fusion-based crop pest and disease classification method and achieved good classification results on 12 pest data sets, with a correct classification rate of 98.2%. Jiao et al. (2022) developed a CNN-based method for the detection of multi-class pests in complex scenarios, and conducted a large number of comparative experiments on the AgriPest21 data set. The results showed that the method could achieve 77.0% accuracy, which was significantly better than other most advanced methods. Mallick et al. (2023) proposed an innovative deep learning-based approach for automatic multi-class mung bean pests and diseases detection and classification, and for each class, the proposed model had an overall pests and diseases detection accuracy of 93.65%. Although the above studies achieved good results, image segmentation and feature extraction in complex and variable background environments were still difficult for their models, the number of experimental samples was limited, and the crop pest and disease recognition models they established were unstable, which indicates that there is still a large gap between research and practical application.

With the development of artificial intelligence technology, researchers have started to detect and study crop pests and diseases with the help of deep learning. Deep learning can automatically, efficiently and accurately extract object features from a large number of crop pest and disease images, thus making up for the shortcomings of traditional manual recognition and enabling crop pest and disease image recognition. Deep learning-based image object detection techniques have enabled great advances, and at present, two main detection methods have been developed. One category is object detection based on deep convolutional networks with region proposal, and the representative models are Fast Region-based Convolutional Neural Network (R-CNN) (Ross, 2015), Faster R-CNN (Ren et al., 2015), and Mask R-CNN (He et al., 2017). Among these models, Faster R-CNN is unique in abandoning the traditional sliding window and selective search methods and instead generates detection boxes directly using a region proposal network (RPN), which greatly improves the detection box generation speed. Another category is object detection based on deep convolutional networks with regression computation, and the representative models include You Only Look Once (YOLO) (Redmon et al., 2016), and single-shot detector (SSD) (Liu et al., 2016). Of the different models in the YOLO series, YOLOv5 uses the PyTorch framework and is user-friendly, not only making it easy to configure the environment but also enabling very fast training of the model. Moreover, it has very good performance in detecting smaller objects. SSD integrates the YOLO concept of fast detection, offers the advantages of RPN that are found in Faster R-CNN, and improves the handling of multi-size objects, which is to say it does not rely solely on the top-level feature map for prediction.

Currently, the research on agricultural object detection for both fruit detection and pest and disease recognition is increasingly turning to deep learning. Shen et al. (2018) used Faster R-CNN to extract regions in images that might contain insects and to classify the insects in these regions, and its mean average precision reached 88%. Liu and Wang (2020) proposed a method for early recognition of tomato leaf spot based on the MobileNetv2-YOLOv3 model, and the results showed that in all test sets, the F1 scores and average precision (AP) values were 93.24% and 91.32%, respectively, and the average IOU value was 86.98%. Chu et al. (2021) developed a novel suppressed Mask R-CNN for apple detection, and the network they developed had an F1 value of 0.905 and a detection time of 0.25 seconds per frame on a standard desktop computer, which were better than the values for state-of-the-art models. Wang X. W. et al. (2021) proposed an improved object detection algorithm based on YOLOv3 to address the problem of the complex background in early stage images of tomato pests and diseases in natural environments; this model enhanced the recognition of pests and diseases, with an average recognition accuracy of 91.81%. Li. et al (2021) proposed a detection method named Lemon-YOLO (L-YOLO) to improve the accuracy and real-time detection of lemons in natural environments. The experimental results show that the AP value and FPS value of the proposed L-YOLO on the lemon test set are 96.28% and 106, respectively 5.68% and 28 higher than that of YOLOv3. Zhang et al. (2021) first developed a synthetic soybean leaf disease image data set, and then designed a multi-feature fusion Faster R-CNN (MF^3^ R-CNN) to detect soybean leaf disease in complex scenes, obtaining the best average precision of 83.34% in the actual test data set. Sun et al. (2021) proposed a mobile-based detection model, Mobile End AppleNet (MEAN)-SSD, for the real-time detection of apple leaf diseases on mobile devices that can automatically extract apple leaf spot features and detect five common apple leaf spots. Qi et al. (2022) proposed a squeeze-and-excitation (SE)-YOLOv5-based object detection model to recognize tomato virus disease. The trained network model was evaluated on a test set, and its mean average precision reached 94.10%. Zhao et al. (2022) proposed a new Faster R-CNN architecture and constructed a strawberry leaf, flower and fruit data set. The results showed that the model was able to effectively detect healthy strawberries and seven strawberry diseases under natural conditions with a mAP of 92.18% and an average detection time of only 229 ms. Liu et al. (2022) proposed a tomato pest identification algorithm based on an improved YOLOv4 fusion triple attention mechanism, and the proposed algorithm was tested on the established data set with an average recognition accuracy of 95.2%. Ahmad et al. (2022) implemented an automated system in the form of a smartphone IP camera for pest detection from digital images/video based on eight YOLO object detection architectures, and the results showed that the YOLOv5x architecture achieved the highest mAP (98.3%) at real-time inference speed and could correctly recognize 23 pests in 40.5 ms. The models presented in these studies can achieve fruit detection as well as accurate classification and recognition of pests and diseases; however, most of the existing studies on models for the recognition of crop pests and diseases focus on recognition of the pests themselves, but *A. gossypii*, due to its small size, large quantities and dense accumulation on the undersides of leaves, is a pest that is difficult to identify directly.

Therefore, utilizing the different infestation symptoms cotton leaves exhibit when infested by A. gossypii and determining the severity of A. gossypii occurrence through the features of leaf infestation is an alternative approach. In this study, the level of *A. gossypii* infestation was determined by creating a model that can assess the symptoms in cotton leaves caused by *A. gossypii* infestation. Images of *A. gossypii* infestation in the field environment were quickly acquired using smartphones, and then the data were annotated to construct four types of data sets: level 0, level 1, level 2 and level 3. On this basis, three different *A. gossypii* infestation recognition models based on Faster R-CNN, YOLOv5 and SSD were constructed, and the test results of the three models were compared and analyzed to select the optimal *A. gossypii* infestation recognition model to deploy it to android mobile side, which provides a fast, convenient and low-cost method for *A. gossypii* infestation monitoring. The infestation class recognition model of A. gossypii established in this study can provide technical support for prediction forecast and precise prevention and cure of A. gossypii, which will enhance the utilization rate of pesticides in the field, reduce the cost of agricultural production and enhance the yield and quality of cotton. Afterwards, it will continue to be deployed to the spraying machinery, striving to achieve simultaneous identification and precise prevention and cure as soon as possible.

## 2 Materials and methods

### 2.1 Experimental design

The experiment was conducted in 2018, 2019, and 2022 at the Korla Experimental Station of the Institute of Plant Protection, Chinese Academy of Agricultural Sciences (41°44′59″N, 85°48′30″E). The experimental station is located in Heshilike Township, Korla City, Bayingol Mongolian Autonomous Prefecture, Xinjiang, China, which is located in the central part of Xinjiang and on the northeastern edge of the Tarim Basin, near the Tianshan Branch to the north and the Taklimakan Desert to the south. Cotton is the main crop grown in this area, and is cultivated with large-scale and simple cropping structures. *A. gossypii* is the main cotton pest in the region, and its peak season occurs from late June to early July (Lu et al., 2022). Experimental plots with severe occurrence of *A. gossypii* were selected in the field for data acquisition. No pesticides were applied to suppress the population growth of this pest during the experiment. The cotton crops selected for the test were the experimental cultivars ‘Zhongmiansuo49’ and ‘Xinluzhong66’ from the Cotton Insect Group of the Institute of Plant Protection, Chinese Academy of Agricultural Sciences. The cotton was sown in mid to late April with a film mulching cultivation mode, and with spot sowing on the film. Standard water and fertilizer management was carried out through drip irrigation under the film.

### 2.2 Data acquisition

Cotton image data were collected at the Korla Experimental Station of the Institute of Plant Protection, Chinese Academy of Agricultural Sciences in 2018, 2019 and 2022 (**Table 1**). The collection dates for 2018 and 2019 were from late June to mid-July, and the collection dates for 2022 were from early June to early July. The collections were made on sunny days and with low light intensity to avoid image overexposure. To allow the model to learn more features of *A. gossypii* infestation during training, multiple smartphones were used to acquire the cotton images. Image data acquisition was conducted in 2018 and 2019 with the HUAWEI Nova, OnePlus7pro, iPhone 8 Plus, and Mi Note 3 smartphones and in 2022 with the iPhone 8 Plus, iPhone 12, iPhone 13, iPhone XR, and Redmi 5 Plus smartphones. The image acquisition method was overhead vertical shooting. The researchers stood next to the cotton plants with mobile equipment in hand and vertically shot images of the cotton seedlings from a vantage of 1.2-1.5 meters. The data acquired in 2018 and 2019 were used for training, validation, and testing of the *A. gossypii* infestation recognition model, and the cotton images collected all featured the ‘Zhongmiansuo49’ cultivar. In 2022, in addition to photographing plants of the ‘Zhongmiansuo49’ cultivar, some images of plants from the ‘Xinluzhong66’ cultivar were also collected for testing the *A. gossypii* infestation level recognition model detection capabilities on images of other cultivars.

**Table 1:** Summary of data acquisition characteristics.

Cotton cultivars	Device	Rear camera pixels (million)	Image resolution	Aperture Value	Focal length (mm)
‘Zhongmiansuo49’	HUAWEI Nova	12	4032 × 3016 3016 × 3016	f/2.2	4
	OnePlus7pro	48	4000 × 3000 4608 × 3456	f/1.6 f/2.2	5 2
	iPhone 8 Plus	12	3024 × 4032	f/1.8	4
	Mi Note 3	12	3016 × 4032	f/1.8	4
‘Zhongmiansuo49’	iPhone 12	12	3024 × 4032	f/1.6	4
	iPhone 13	12	3024 × 4032	f/1.6	5
	iPhone XR	12	3024 × 4032	f/1.8	4
	iPhone 8 Plus	12	3024 × 4032	f/1.8	4
‘Xinluzhong66’	iPhone XR	12	3024 × 4032	f/1.8	4
	Redmi 5 Plus	12	3000 × 4000	f/2.2	4

### 2.3 Image processing

**Figure 1:**
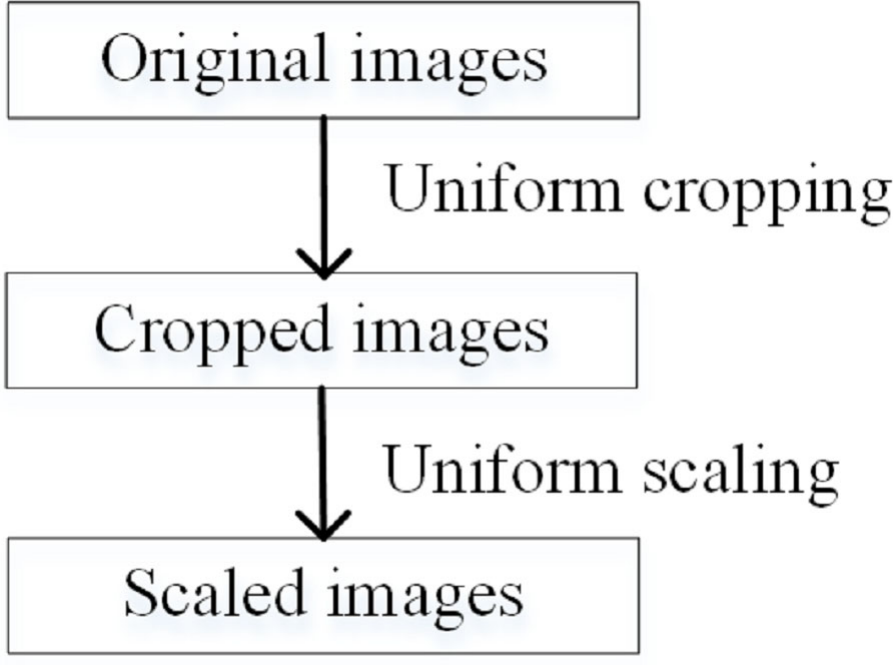
Data preprocessing flow chart.

Directly inputting original images into a model for training can interfere with training by causing problems such as taking up a large amount of memory on the device, slowing down the training speed of the model, and causing memory overflow. Therefore, the original images must be preprocessed. When inputting images into the model, the image formats are adjusted to a certain size. To prevent soil and crop backgrounds from interfering with object detection, the original image is cropped to remove redundant information such as soil from the image. To crop the images, we first started from the center of each original cotton image, cropping the image to 3000×3000 and uniformly adjusting the image resolution to 1024×1024. Then the data were annotated. The data preprocessing process of this paper is shown in **Figure 1**.

### 2.4 Data set construction

**Figure 2:**
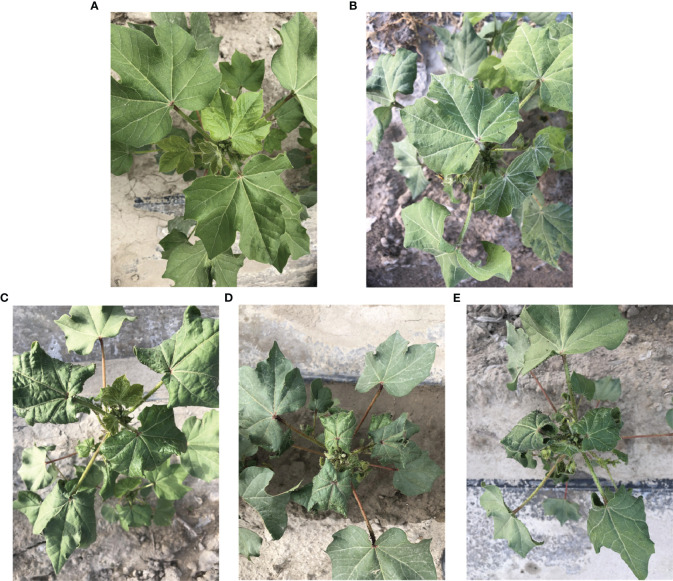
Example of acquired images: **(A)** level 0, **(B)** level 1, **(C)** level 2, **(D)** level 3, **(E)** level 4.

Because the training data of the object detection model need to be manually labeled, LabelImg (Tzutalin, 2015) was chosen as the image labeling tool in this experiment. Examples of acquired images for this experiment are shown in **Figure 2**. When labeling the image data, the *A. gossypii* infestation grading standard referred to the national grading standard (GB/T 15799-2011, 2011) (**Table 2**). Since the level of infestation suffered by cotton leaves at the seedling stage rarely reaches level 4, individual level 4s were classified as level 3s when conducting data annotation to avoid serious imbalance in the data set. The annotation entailed using rectangular boxes to annotate the cotton images, and according to the grading standard in **Table 2**, the leaves in the central region of the cotton plant were annotated as level 0, level 1, level 2 and level 3 according to their different morphological characteristics, and the generated annotation files were all saved as XML files in PASCAL VOC format. The annotated images included the images collected by the various collection devices in 2018 and 2019, and a total of 3051 ‘Zhongmiansuo49’ cotton images were annotated, including 295 from the HUAWEI Nova, 1237 from the OnePlus7pro, 1270 from the iPhone 8 Plus and 249 from the Mi Note 3.

**Table 2:** *Aphis gossypii* Glover infestation grading standards.

Infestation level	Infestation description
0	No aphids, spreading leaf blades.
1	There are aphids, but the leaves are not damaged.
2	There are aphids, and the most severely damaged leaves are crinkled or slightly rolled, nearly semicircular.
3	There are aphids, and the most heavily damaged leaves are curled up in a semicircle or more than semicircle, and are arc-shaped.
4	There are aphids, and the most heavily damaged leaves are completely curled and appear spherical.

To balance the number of labels, we also performed a left-right flip operation on 188 annotated images from the iPhone 8 Plus and 151 annotated images from the Mi Note 3, resulting in 3390 annotated images. After data enhancement by flipping up and down, adding Gaussian noise and changing image brightness, the number of images was 16,950, and 16,950 annotated images were used as the data set for this experiment. The data set was first divided into a training validation set and a test set at a ratio of 9:1, and then the training validation set was divided into a training set and a validation set at a ratio of 9:1. The final training set was 13,729, the validation set was 1,526, the test set was 1,695, and the number of labels for each infestation level was 10,554 for level 0, 10,854 for level 1, 11,066 for level 2, and 11,025 for level 3.

### 2.5 Model construction

In this study, three classical object detection models, Faster R-CNN, YOLOv5 and SSD, were chosen to conduct the study of *A. gossypii* infestation level recognition.

**Figure 3:**
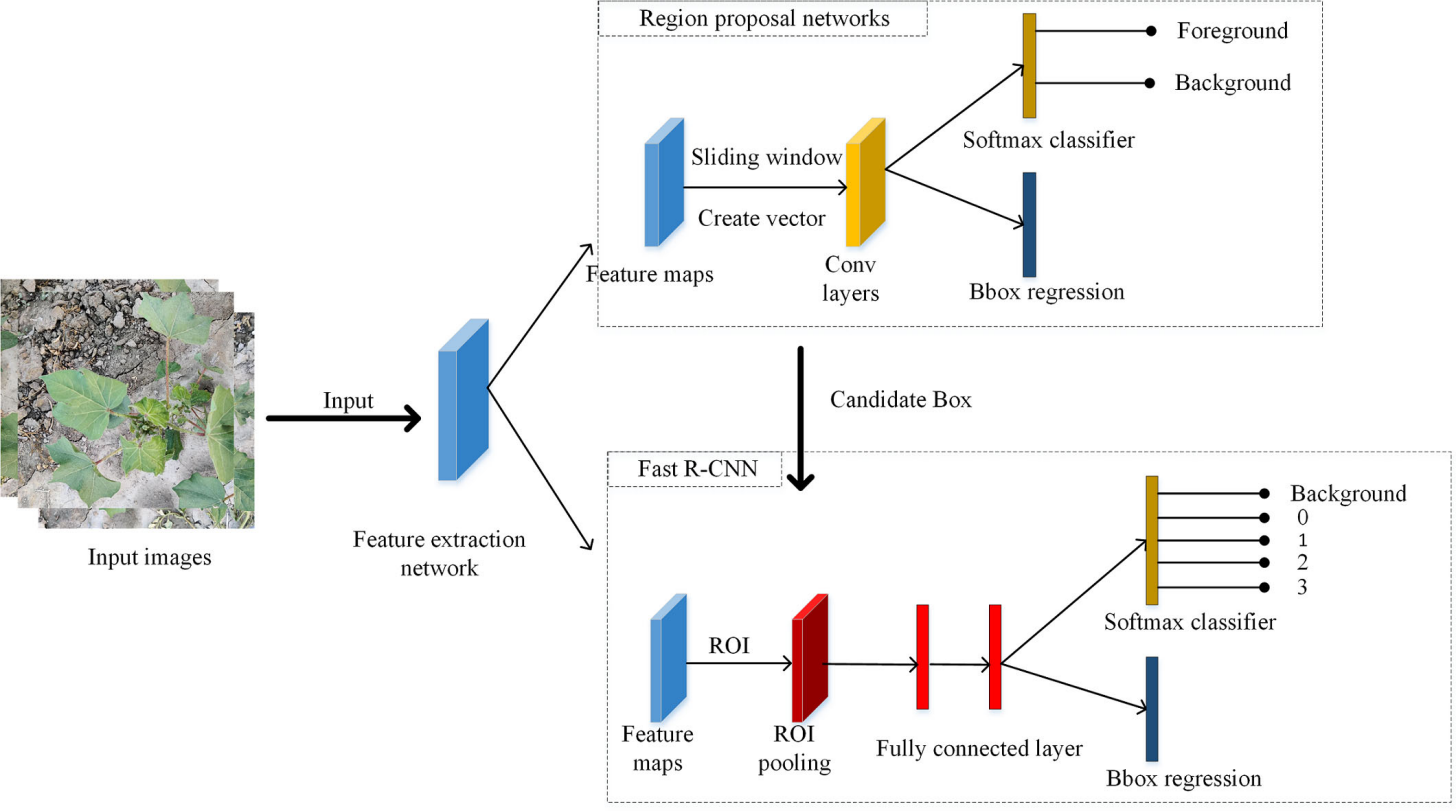
Faster R-CNN model structure.

The structure of the Faster R-CNN (Ren et al., 2015) model is shown in **Figure 3**. The model first used the feature extraction network to extract the feature map of each input cotton image, which was shared by the subsequent RPN with the Fast R-CNN network. RPN performs the binary classification task through a softmax classifier, determines whether the anchor belongs to the foreground or background, and obtains the candidate box position through anchor regression. The Fast R-CNN synthesizes the information from the feature maps and candidate boxes, determines the category to which the foreground belongs, and generates the exact location of the final detection box.

**Figure 4:**
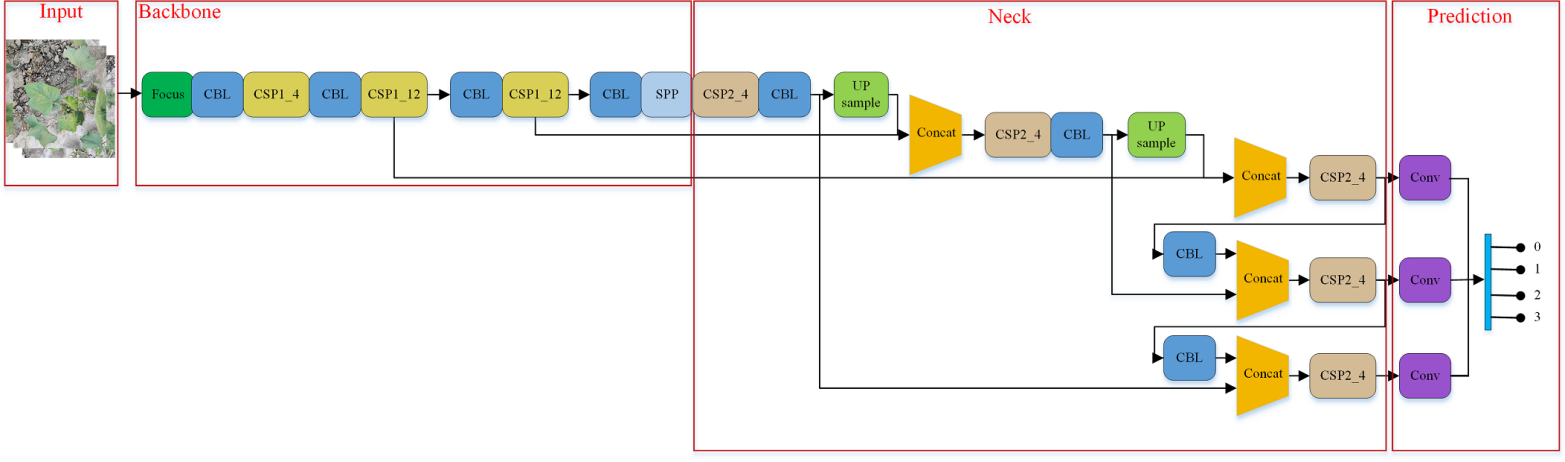
YOLOv5 model structure.

The structure of the YOLOv5 (Jocher, 2020) model is shown in **Figure 4**. The network structure of the model is divided into four parts according to the processing stage: input, backbone, neck and prediction. The input part completes basic processing tasks such as data enhancement, adaptive image scaling and anchor box calculation. The backbone network mainly uses a common spatial pattern (CSP) structure to extract the main information from the input samples for use in subsequent stages. The neck part uses feature pyramid network (FPN) and path aggregation network (PAN) structures and uses the information extracted from the backbone to enhance feature fusion. The prediction component makes predictions and calculates the value of each loss. YOLOv5 has four model styles, s, m, l and x. They have the same network structure, and only the depth and width of the models are different.

**Figure 5:**
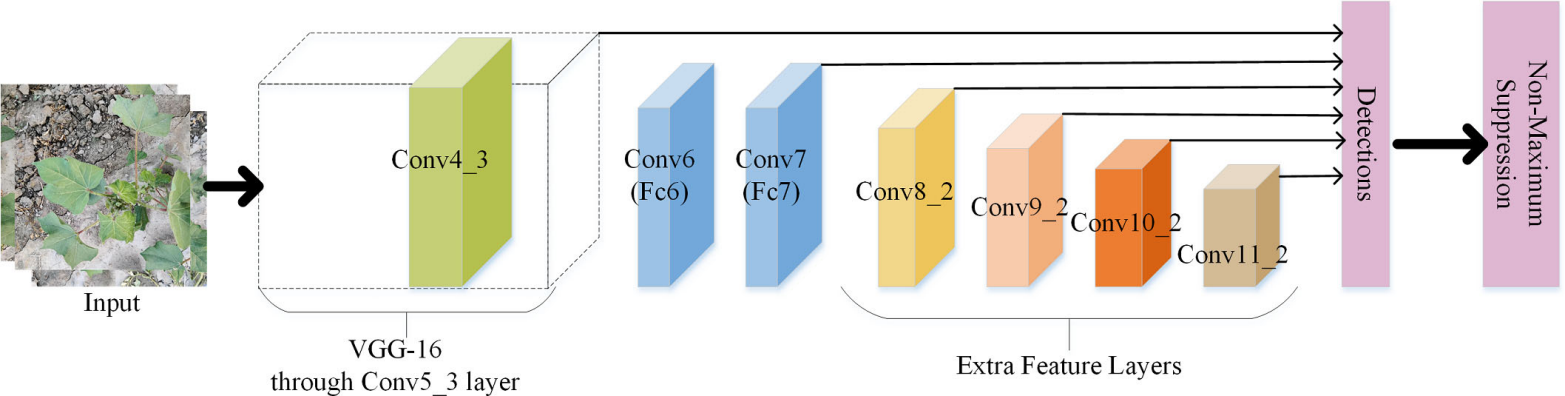
SSD model structure.

The structure of the SSD (Liu et al., 2016) model is shown in **Figure 5**. The model inputted cotton images into the backbone network, which could obtain feature maps of different sizes from the pretrained base network, and feature maps of six convolutional layers of different sizes, Conv4_3, Conv7, Conv8_2, Conv9_2, Conv10_2, and Conv11_2, were the output. Six default candidate boxes with different aspect ratios were constructed from each pixel point of these feature maps and then detected and classified separately to generate multiple initial eligible default candidate boxes. Finally, the nonmaximum suppression method was used to screen out the eligible candidate boxes to generate the final set of detected boxes, that is, the *A. gossypii* infestation level.

### 2.6 Experimental environment

On the basis of the construction model, the hardware environment was as follows: graphics processor, NVIDIA A 100-PCIE-40 GB; CUDA Cores, 6912; total memory, 4060 MB; and memory interface, 5120-bit. The software environment included PyCharm (2020.3, JetBrains, Prague, Czech), Linux (Ubuntu 20.04.4 LTS, Linus Benedict Torvalds, Helsinki, Suomi), Python (Python 3.8.12, Python Software Foundation, State of Delaware, USA), PyTorch (PyTorch 1.10.2 or PyTorch 1.10.0+CUDA 11.3, Facebook AI Research, California, USA), and Android Studio (2021.2.1, Google, California, USA).

### 2.7 Evaluation metrics

To quantitatively analyze the performance of a detection algorithm, researchers have formulated many evaluation metrics, each reflecting different aspects of the performance to some extent. The object detection performance evaluation metrics used in this experiment were Precision (Yang, 1999). Recall (Yang, 1999), Accuracy, Average Precision (AP), mean Average Precision (mAP), and Frames Per Second (FPS).

For each category, a curve can be drawn according to the precision and recall rate, and the AP value is the area under the curve. The mAP value is the average value of AP for each category. The classification and localization ability of the object detection model is its main performance representation, and the mAP value is its most intuitive expression. The larger the mAP value is, the higher the precision of the model; the detection speed represents the computational performance of the object detection model and is represented by the FPS value, and the larger the FPS value is, the better the detection speed of the algorithm model (Xu et al., 2021).

The formula for calculating each evaluation metric is shown below:


(1) $$P=\frac{TP}{TP+FP}\;\;$$

(2) $$R=\frac{TP}{TP+FN}\;\;$$

(3) $$Accuracy=\frac{TP+TN}{TP+FP+FN+TN}\;\;\;$$

(3) $$\;\;\;AP=\overset{1}{\underset{0}{\int }}P(R)dR\;\;\;\;\;\;\;\;\;\;\;\;\;\;\;\;\;\;\;\;$$

(4) $$\;\;\;\;mAP=\;\;\;\frac{\sum_{1}^{n}AP}{n}\;\;\;\;\;$$

(5) $$FPS=\;\;\frac{frameNum}{elapsedTime}\;\;$$

Where P is precision, R is recall, TP is positive samples correctly predicted as positive samples, FP is negative samples incorrectly predicted as positive samples, FN is positive samples incorrectly predicted as negative samples, TN is negative samples correctly predicted as negative samples, n is the number of categories, frameNum is number of images, and elapsedTime is detection time.

## 3 Results

### 3.1 Selecting the best model

In this experiment, three models, Faster R-CNN, YOLOv5 and SSD, were selected, and the same data set was used with each model. Since the width and depth of Yolov5x model were the largest among the four model styles, x model was chosen here to participate in the comparison test. Before training, the image resolution was uniformly set to 512×512, the learning rate was set to 0.0005, the iteration rounds were set to 300 rounds, and the batch size of each iteration was set to 16. The training model was saved, and then the model was tested and evaluated with the test set. The AP, mAP, and FPS obtained for the three models’ tests are shown in **Table 3**.

**Table 3:** Precision evaluation of each model for detection of *Aphis gossypii* Glover infestation level.

Model	AP (%)				mAP (%)	FPS
	0	1	2	3		
Faster R-CNN	88.1	86.2	86.8	88.6	87.4	10.44
YOLOv5x	94.6	94.4	96.7	97.3	95.7	61.73
SSD	63.2	59.2	50.8	73	61.5	7.64

As shown in **Table 3**, it can be seen that the mAP value after testing was 87.4% for the Faster R-CNN model, 95.7% for the YOLOv5 model, and 61.5% for the SSD model. Through the test results, it was revealed that the mAP value of the YOLOv5 model for recognizing *A. gossypii* infestation levels was higher than those of the Faster R-CNN and SSD models, which indicates that this model has the highest precision. The YOLOv5 model took the least amount of time to train, at 15.62 hours, under the same conditions that were used with all three models, while the Faster R-CNN and SSD models took 34.32 hours and 33.24 hours to train, respectively. It can also be seen from **Table 3** that the YOLOv5 model had the fastest detection speed, with an FPS value of 61.73, which was much faster than the other two models, indicating that this model has the best detection speed.

In summary, the test results showed that the YOLOv5 model requires the shortest training time and has the highest mAP value and the fastest detection speed, with a mAP value of 95.7% and an FPS value of 61.73. Therefore, this model has the best performance.

### 3.2 Influence of different image resolutions on the performance of the YOLOv5 model

After the comparison of the three models, it was found that the best performance at an image resolution of 512×512 was the YOLOv5 model. To verify the effect of different image resolutions on model performance, two model styles, s and x, in the YOLOv5 model were selected to study the performance of the model at image resolutions of 1024×1024, 640×640, 512×512, 256×256, and 128×128.

**Figure 6:**
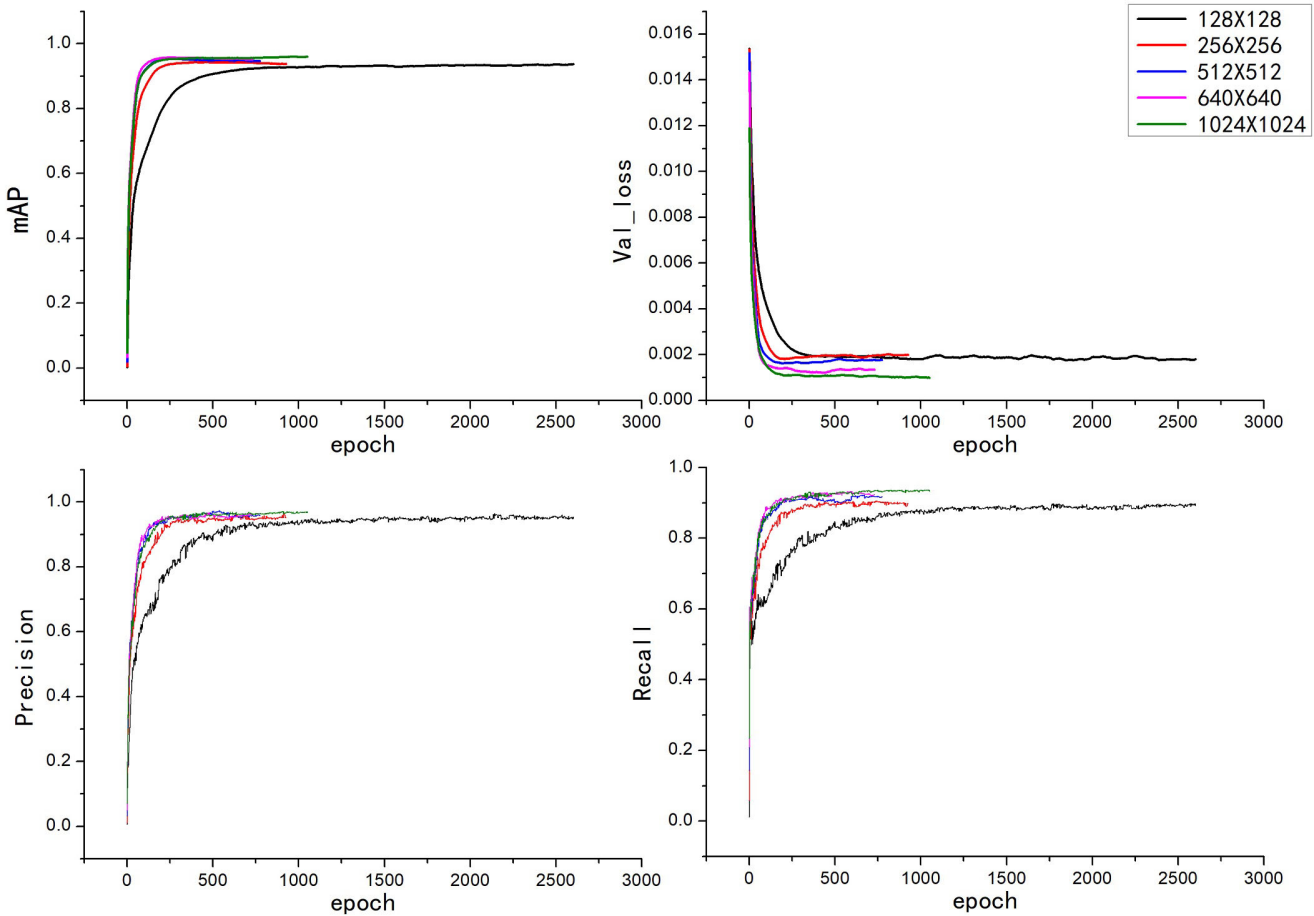
Training results of the YOLOv5x model with different image resolutions.

**Figure 7:**
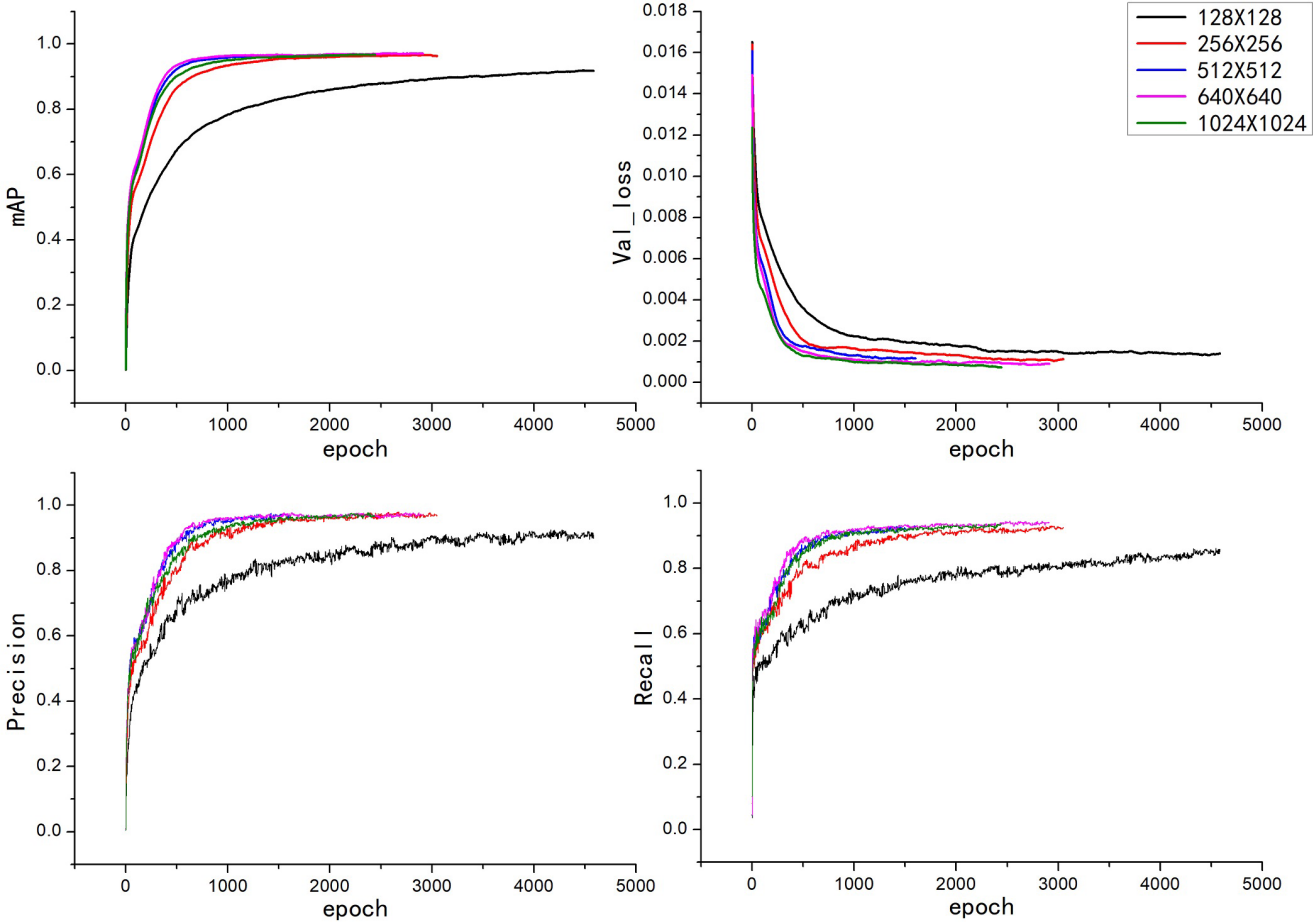
Training results of the YOLOv5s model with different image resolutions.

The data set used in this experiment was the same as the data set used in the construction of the three models, the learning rate was uniformly set to 0.0005, the number of iterations rounds was set to 10,000, the batch size of each iteration was set to 16, and the image resolution was set appropriately for each according to the requirements of the experiment before training. There is an early stop mechanism in the YOLOv5 model; that is, after a certain quantity of training iteration rounds, if the model effectiveness has not improved, then the model training is stopped early. The patience parameter set in this experiment was 100; that is, during the model training, training was stopped if the model effectiveness did not improve within 100 consecutive rounds. The training models with different image resolutions were saved. The training results for YOLOv5x and YOLOv5s are shown in **Figures 6**, **7**. Then, the training models for each image resolution were evaluated with the test set. The test results obtained are shown in **Table 4**.

**Table 4:** Comparisons of the test results of the YOLOv5 model with different image resolutions.

Model	Image size	Train time (hour)	AP (%)				mAP (%)	P (%)	R (%)	FPS
			0	1	2	3				
YOLOv5x	1024×1024	141.55	94.0	95.2	96.6	97.7	95.9	96.9	93.5	34.65
	640×640	44.66	94.2	94.6	96.6	97.7	95.8	96.1	92.7	51.82
	512×512	35.65	93.5	94.4	95.8	96.7	95.1	95.5	92.0	61.79
	256×256	33.48	91.4	92.1	95.2	95.9	93.7	95.2	90.4	76.39
	128×128	89.77	92.2	93.0	95.3	95.8	94.1	95.6	89.9	81.22
YOLOv5s	1024×1024	111.40	95.8	94.7	97.7	97.8	96.5	96.9	93.3	62.71
	640×640	108.27	96.1	95.3	97.7	98.1	96.8	97.8	93.4	71.43
	512×512	52.16	95.1	95.4	97.0	97.9	96.3	95.9	93.1	78.58
	256×256	90.91	95.4	95.9	97.7	97.9	96.7	97.1	92.5	82.56
	128×128	132.21	87.3	92.7	93.3	93.0	91.6	89.5	85.5	92.32

As shown in **Figure 6**, the convergence speed of the YOLOv5x model was the slowest when the image resolution was 128×128, and the convergence speed for several other image resolutions did not differ much. When the image resolution was 1024×1024, the mAP value of the validation set reached 96%, which was the highest mAP value among the five image resolutions, indicating that this was the resolution at which the model had the best train effect.

As shown in **Figure 7**, the convergence speed of the YOLOv5s model was the slowest when the image resolution was 128×128, and the convergence speed of the remaining image resolutions was relatively similar. When the image resolution was 640×640, the mAP value of the validation set reached 97.1%, which was the highest mAP value among the five image resolutions, indicating that this was the resolution at which the model had the best train effect.

Comparing **Figures 6** , **7**, it can be seen that the convergence speed of the YOLOv5s model was significantly lower than that of the YOLOv5x model. Except for at the 128×128 image resolution in the YOLOv5x model, the YOLOv5s model generally had more training rounds than the YOLOv5x model for all image resolutions.

As shown in **Table 4**, it can be seen that in the YOLOv5x model, when the image resolution was 1024×1024, the model took the longest time from training to stopping, at 141.55 hours, and the test set had the highest mAP value, at 95.9% but the lowest FPS value, at 34.65. When the image resolution was 256×256, the model took the least amount of time from training to stopping, at 33.48 hours, and the test set had the lowest mAP value, at 93.7%. When the image resolution was 128×128, the test set had the highest FPS value, at 81.22. Overall, the performance was best when the image resolution was 640×640, as the mAP value for that image resolution was 0.1% lower than the highest mAP value, which was for an image resolution of 1024×1024, but the FPS value was 17.17 higher. In the YOLOv5s model, when the image resolution was 640×640, the test set had the highest mAP value, at 96.8%. When the image resolution was 128×128, the model took the longest time from training to stopping, at 132.21 hours, and the test set had the lowest mAP value, at 91.6%, but the highest FPS value, at 92.32. When the image resolution was 1024×1024, the test set had the lowest FPS value, at 62.71. When the image resolution was 512×512, the model took the least amount of time from training to stopping, at 52.16 hours. Overall, the performance was best when the image resolution was 640×640 because although the FPS value for this image resolution was not the highest, the mAP value was the highest, at 96.8%.

Comparing the YOLOv5x and YOLOv5s models, as seen in **Table 4**, the YOLOv5s model performed better than the YOLOv5x model, both in terms of mAP values and FPS values. The model performed best when the image resolution was 640×640 with a mAP value of 96.8% and an FPS value of 71.43.

### 3.3 Supplementary tests

The best performance of YOLOv5s was found in the previous study comparison, and its authors updated YOLOv8 version in early 2023, so this paper conducts a supplementary experiment to compare the YOLOv5s model in this paper with the latest YOLOv8s model, and its comparison results are shown in **Table 5**.

**Table 5:** Comparison of model test results between YOLOv5s and YOLOv8s.

Model	AP (%)				P (%)	R (%)	mAP (%)	FPS
	0	1	2	3				
YOLOv5s	96.1	95.3	97.7	98.1	97.8	93.4	96.8	71.43
YOLOv8s	96.4	97.1	98.1	98.3	97.6	93.9	97.5	67.75

As shown in **Table 5**, the YOLOv5s model achieves the mAP value of 96.8% and the FPS value of 71.43 on the test set. The YOLOv8s model achieved the mAP value of 97.5% and the FPS value of 67.75 on the test set. Compared to the YOLOv5s model, the mAP value of YOLOv8s improved by 0.7% and the FPS value decreased by 3.68. Although YOLOv8s improved 0.7 percentage points in detection precision over YOLOv5s, the detection speed dropped by 3.68. So all in all, YOLOv5s performed better than YOLOv8s.

### 3.4 YOLOv5 model based on the Android mobile platform

To facilitate real-time detection in the field, the YOLOv5 model with the best test results was deployed to Android mobile. Based on the test results presented in **Table 4**, we chose to deploy the trained YOLOv5s models of each image resolution in **Figure 7** to the Android mobile platform one by one and compare their detection effects. The model files saved after training were first converted into the corresponding files, then the code was debugged in Android studio and finally deployed to the Android mobile platform, and the test machine used in this experiment was a Redmi 5 Plus (Android Version:8.1.0; GPU Model: Adreno 506; Operating Memory:3GB; Storage Capacity:32GB). After deployment, the Android Package Kit (APK) for each resolution had an application size of 1.62 GB and required 16.38 KB of user data.

**Figure 8:**
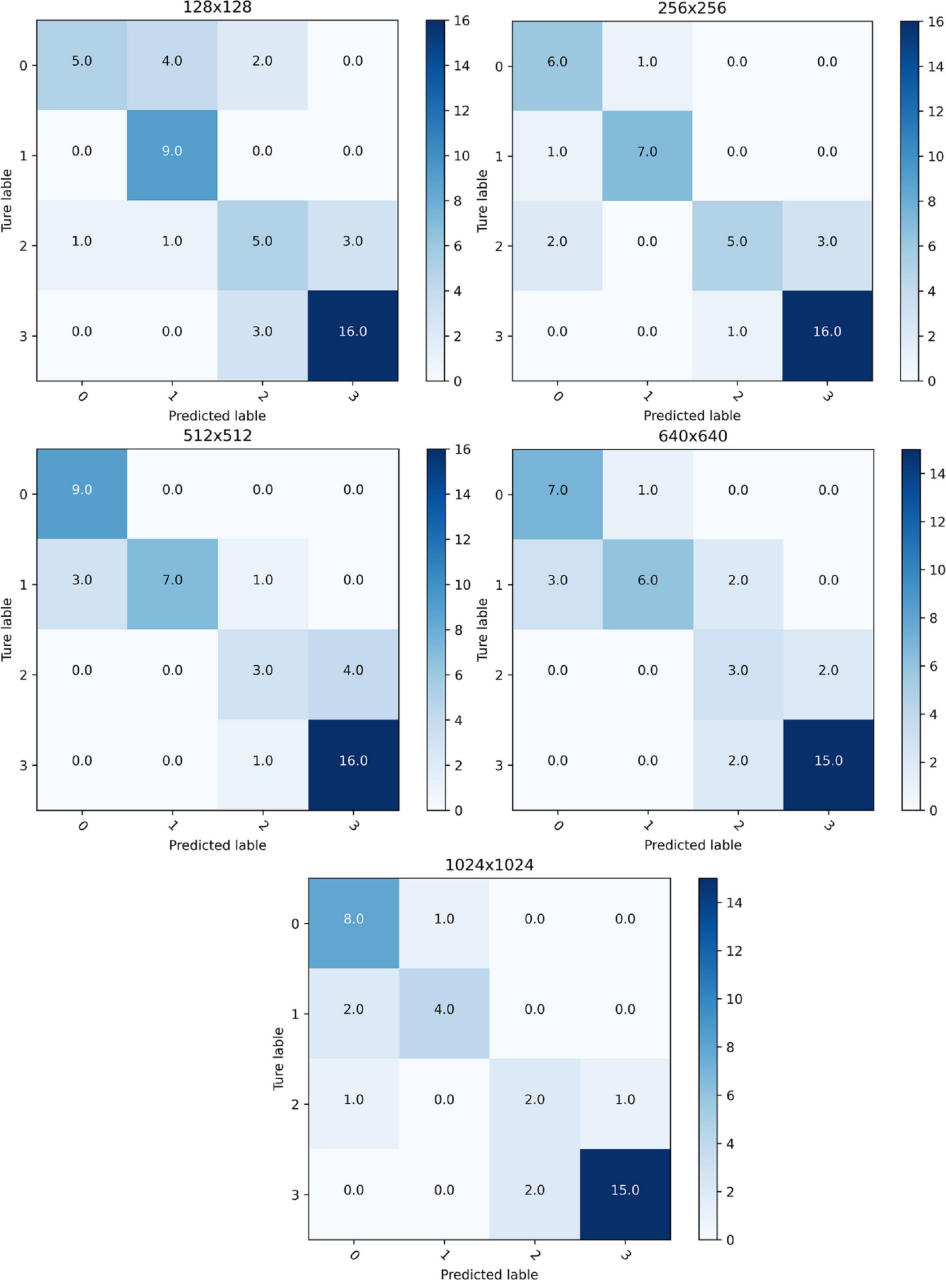
Confusion matrix of detection results for different image resolutions.

In this experiment, 20 original images of ‘Zhongmiansuo49’ cultivar plants from the image data collected in 2018 and 2019 were reselected for mobile platform detection, and the results of the mobile platform detection for different image resolutions are shown in **Figure 8**; **Table 6**. The total number of leaves detected at different resolutions were between 35 and 50. The confusion matrix indicates whether the model confounds different categories by comparing the actual infestation grades of the blades in the mobile platform test data with the predicted infestation grades. As shown in **Table 6**, when the image resolution was 256×256, the accuracy of the mobile platform was the highest, at 81.0%, and the detection speed was also the fastest, with an FPS value of 6.98. Therefore, the detection of the mobile platform is the best at this image resolution.

**Table 6:** Detection results of the mobile platform at different image resolutions.

Image size	Accuracy (%)	FPS
1024×1024	80.6	6.76
640×640	75.6	6.53
512×512	79.5	6.79
256×256	81.0	6.98
128×128	71.4	6.87

### 3.5 Evaluation of model performance in the field environment

**Figure 9:**
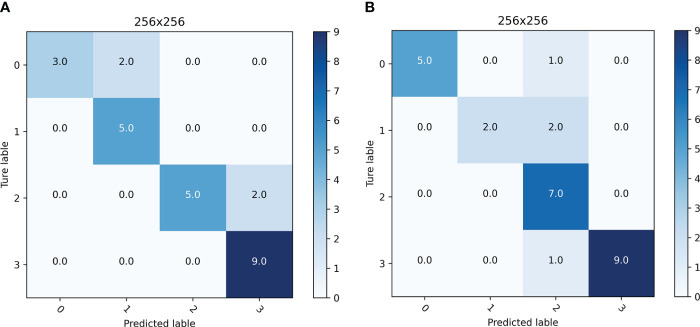
Confusion matrix of the detection results for infested leaves of two different cultivars: **(A)** ‘Zhongmiansuo49’, **(B)** ‘Xinluzhong66’.

**Figure 10:**
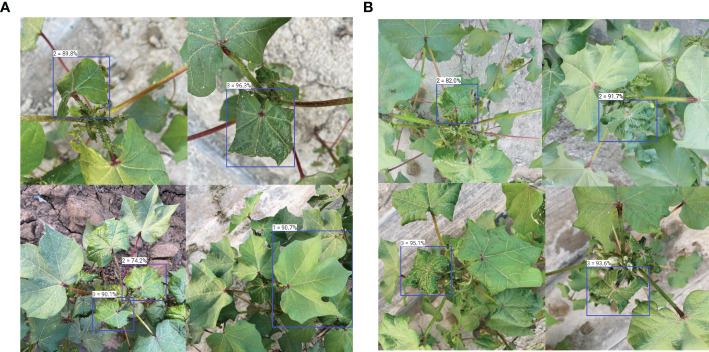
Examples of detection results for infested leaves of two different cultivars **(A)** ‘Zhongmiansuo49’, **(B)** ‘Xinluzhong66’.

Separately selecting 20 cotton images from the ‘Zhongmiansuo49’ and ‘Xinluzhong66’ cultivars from the new data collected in 2022, the accuracy and usefulness of the YOLOv5 model, which had been successfully deployed on the Android platform, were evaluated. The test results are shown in **Figure 9**; **Table 7**, and an example of the detection results is shown in **Figure 10**.

**Table 7:** Mobile platform detection results for infested leaves of two different cultivars.

cultivars	Accuracy (%)	FPS
‘Zhongmiansuo49’	84.6	8.61
‘Xinluzhong66’	85.2	8.19

By comparing the actual infestation levels and predicted infestation levels of the leaves in the mobile platform test data (**Figure 9**), it can be seen that the adjacent levels are easily confused with each other, and that level 3 is confused the least, probably because the leaf features at levels 0 and 1 are similar, and that the curl of the leaves at level 3 is very obvious and easy to judge. As presented in **Table 7**, the mobile-side detection accuracy for infested leaves of the ‘Zhongmiansuo49’ cultivar was 84.6%, and the detection speed FPS value was 8.61; the mobile-side detection accuracy for infested leaves of the ‘Xinluzhong66’ cultivar was 85.2%, and the detection speed FPS value was 8.19. As shown in **Table 7**, the accuracy and usefulness of the YOLOv5 model deployed on the Android platform are relatively ideal and can provide a more convenient and faster means for field investigators to use the model.

## 4 Discussion

For *A. gossypii* infestation class identification, other researchers have more often studied multispectral (Zeng et al., 2021; Fu et al., 2022) and hyperspectral (Feng and Liu, 2020) cotton images collected by unmanned aerial vehicle (UAV), while our study was based on cotton images collected by smartphones. The methods of the two approaches are different; the cotton images collected by UAV can qualitatively determine the degree of *A. gossypii* infestation at a macro level, with the degree of *A. gossypii* infestation reflected by the spectral curve characteristics of the cotton canopy, while the cotton images taken by smartphones in this paper can quantitatively determine the degree of *A. gossypii* infestation of a single cotton plant, with more accurate results. The object detection models constructed in this study were all able to recognize *A. gossypii* infestation levels, the mAP value for the best YOLOv5 model reached 96.8%, and its FPS value reached 71.43.

After comparing the test results of the three models, it was found that the mAP values of the SSD model were much lower than those of the other two models. By reviewing the model debugging details, it appears that this result may be due to the use of a static learning rate or an excessively high learning rate setting. To compare the three models, the learning rate must be uniformly set to a static learning rate; thus, the same learning rate was set for all three models in this paper.

The objectives of this study were to compensate for the shortcomings of traditional *A. gossypii* survey methods, to enhance the efficiency of *A. gossypii* infestation detection and to expand the application of object detection algorithms. Most of the current field applications of pesticides in production are quantitative, which can lead to overuse of pesticides, thus increasing production costs and simultaneously causing environmental pollution. The best model identified in this study achieves real-time and rapid recognition of the degree of infestation of *A. gossypii* to help mitigate the abovementioned problem. Successfully deploying the model to the mobile platform, and subsequently deploying the model to plant protection UAVs and pesticide application tractors to establish a precision pesticide application technology system for controlling *A. gossypii* infestation will provide technical support for precise pesticide application, which will enhance the utilization rate of pesticides, reduce the cost of agricultural production and improve the ecological conditions of the environment.

## 5 Conclusion

This study used smartphones to quickly and easily collect images of cotton seedlings. Three classical object detection models to achieve fast recognition of *A. gossypii* infestation levels were constructed. The three models were tested, and it was found that the YOLOv5 model had the best performance, with mAP values 8.3% and 34.2% higher than those of the Faster R-CNN and SSD models, respectively, and FPS values that were 51.29 and 54.09 higher than those of the Faster R-CNN and SSD models, respectively, with higher precision and faster detection speed. Based on further testing guided by these results, it was determined that the comprehensive performance of the YOLOv5s model was better than that of the YOLOv5x model at different image resolutions, and that the best performance was achieved when the image resolution was 640×640. And the comparison with the latest YOLOv8s showed that the YOLOv5s performed better than the YOLOv8s. Regarding detection speed and mobility, we successfully deployed the YOLOv5s model to the Android mobile platform, and after testing, it was found that the detection effect on mobile was the best when the image resolution was 256×256. The accuracy was 0.4%, 5.4%, 1.5%, and 9.6% higher at this image resolution than at several other resolutions, and the FPS values were 0.22, 0.45, 0.19, and 0.11 higher than at the other image resolutions, respectively. In addition to images from the ‘Zhongmiansuo49’ cultivar, the model in this study was also used tested on images from the ‘Xinluzhong66’ cultivar, with a final accuracy of 85.2% and an FPS value of 8.19, indicating that the *A. gossypii* infestation level recognition model presented in this paper can be used for the detection of this pest in other cotton cultivars. The *A. gossypii* infestation level recognition model established in this study can provide a faster and more convenient technical mean for *A. gossypii* infestation monitoring, preventing the outbreak of the insect pest in advance and achieving precise prevention and cure, which in turn can help enhance the yield and quality of cotton.

## Data availability statement

The raw data supporting the conclusions of this article will be made available by the authors, without undue reservation.

## Author contributions

All authors have made significant contributions to this research. XX, YL and HQ conceived the ideas and designed the methodology. JS, QH and YC conducted the experiments. XX and JS performed the data acquisition and processed and analyzed the data. LL, TS, RD, YL and HQ performed the supervision. XX, JS and HQ wrote and edited the paper. All authors discussed and wrote the manuscript and gave final approval for publication. YL and HQ acquired the funding.
